# Predatory Publishing and Turkey

**DOI:** 10.4274/balkanmedj.galenos.2019.2019.4.001

**Published:** 2019-07-11

**Authors:** Zafer KOÇAK

**Affiliations:** 1 Department of Radiation Oncology, Trakya University School of Medicine, Edirne, Turkey

Since the beginning of this century, widespread use of the internet and some weaknesses of the existing system have changed the scientific publishing model (1). This new model, wherein authors pay publishers for their services, is termed “open access”. In the last decade, the number of open access journals and the articles they publish have increased rapidly, and some journals have gained a high level of scientific prestige in their field. Despite the rapid growth of the open access market, its share of the whole market remains small. According to an estimation by Delta Think (Carlin 2017), open access accounts for 20%-22% of market volume and 5%-9% of market value (2).Since the beginning of this century, widespread use of the internet and some weaknesses of the existing system have changed the scientific publishing model (1). This new model, wherein authors pay publishers for their services, is termed “open access”. In the last decade, the number of open access journals and the articles they publish have increased rapidly, and some journals have gained a high level of scientific prestige in their field. Despite the rapid growth of the open access market, its share of the whole market remains small. According to an estimation by Delta Think (Carlin 2017), open access accounts for 20%-22% of market volume and 5%-9% of market value (2).

This system provides rapid and open access to scientific information and data. However, some publishers do not hesitate to use this new model for unethical purposes and have begun to accept and publish scientific articles without a genuine peer-review process. Jeffrey Beall and his blog (Scholarly Open Access) played a key role in unveiling these unethical publishers and journals. He became interested in this topic when he received a spam e-mail in 2009. In 2010, he coined the term “predatory open access publishing” and has developed a blacklist of “predatory” journals (Beall’s list). According to the view published in Nature, Beall described these predatory publishers as follows: they publish counterfeit journals to exploit the open access model, in which the author pays (3). Two striking experiments by Bohannon (4) and Sorokowski et al. (5), published in Science and Nature, demonstrated the widespread and alarming nature of this type of predatory publishing.

A study by Xia et al. (6) examined author profiles from some of these “predatory” journals as well as from groups of more recognizable open access journals. They reported that authors who publish in predatory journals are mostly from developing countries, especially India, Nigeria, and some African and Middle Eastern countries. So, the problem seems to be particularly true for authors who find it difficult to publish in English journals. Most of them are young writers in developing countries whose native language is not English. With this editorial review, we tried to shed light on the place occupied by Turkey in this dishonest publishing practice.In the literature review, three articles 2018 gave us some indications of the involvement of Turkey and Turkish scientists in predatory publishing practices. Demir (7) explored the addresses of predatory journals and authors, the identities of the editors, and the authors’ reasons for publishing. Akça and Akbulut (8) analyzed the predatory journals originating from Turkey on Beall’s list. Another study investigated the pros and cons of the new financial support policy for Turkish researchers (9).

Demir (7) carried out an important study investigating the characteristics of all journals included on Beall’s list of standalone journals with accessible websites (832 journals). He was looking for answers to these four questions: What are the foundation and administration locations of the journals on Beall’s standalone journals list? Which countries’ researchers publish more frequently in these journals? Who are the editors of these journals? and Why do Turkish researchers publish in these journals? In this study, according to the IP/WHOIS contact locations, the journals were primarily located in India (62.0%), followed by the US (12.6%) and Turkey (3.9%). In 2017, the highest number of researchers publishing in these journals were from India (10.4%), Nigeria (4.8%), Turkey (3.7%), the US (3.5%), and China (3.5%). The editors of these journals were a cause for concern in Turkey, as Turkey was again in the top three. The editors of these journals were primarily located in India (57.9%), followed by the US and Turkey. Almost 90% of editors were researchers at universities in various countries. Another purpose of this study was to determine the reasons why researchers published their studies in these journals. In addition to the known reasons (academic promotion, lack of awareness, “publish or perish”, etc.), they reported that the academic incentive allowance system was one of the important factors.

Akça and Akbulut (8) examined the journals on Beall's list with Turkish addresses. Beall's list was reviewed in December 2017, and 1268 of 1319 journals were included in the study. They found that 55% (693) of the journals on the list were from India. Interestingly 3.2% (41) of the journals originated from Turkey. With this ratio, Turkey was second on the list after India. One of the most interesting findings of the study was that 30% of these journals originated in universities. These are a cause for deeper concern in Turkey. Seven of these 41 journals have been published since 2015.

The above two studies stated that the new incentive allowance system effective since 2016, known as the ex-post funding system, could be one of the factors that led Turkish researchers to publish in predatory journals. Therefore, it was worth investigating whether the initiation of the academic incentive allowance system has an impact on this. Demir's (9) work partially shed light on this subject.

The study sought to answer two questions: 1. What were the downsides of the ex-post funding system to academic publishing among Turkish researchers? and 2. What were the contributions of the ex-post funding system to academic publishing? (9). He scanned the journals on Beall’s list to determine the number of articles published by Turkey over 2 years before (2014-2015) and after (2016-2017) implementation of the ex-post funding system. It was reported that after the ex-post funding system, the number of articles published in predatory journals increased significantly (455 in 2014-2015 vs 1045 in 2016-2017, p=0.04). Moreover, the number of papers presented at questionable conferences was also increased (49 in 2014-2015 vs 408 in 2016-2017) after the ex-post funding system. He noted that this 130% increase in articles published in predatory journals and 732% increase in papers presented at questionable conferences could not be explained by an increase in the number of researchers. Furthermore, he concluded that the number of publications in potentially fake journals and questionable conferences increased after the ex-post funding system was implemented.

As shown in the data given above (7,8,9), when one examines the number of journals, publications, and editors from Turkey on Beall’s list, Turkey is among the top three countries. This shows the seriousness of the issue in our country. Fortunately, some necessary steps have already been taken by the Turkish Council of Higher Education. However, additional measures that can be taken individually and institutionally will be useful to raise awareness of this fraudulent publication practice and discourage researchers from submitting manuscripts to predatory journals (10). These recommendations are shown in Table 1 (11).

Since 2016, under the new incentive allowance system implemented in Turkey, researchers are paid an incentive for articles published in any journal and for papers presented at any international conference. A new arrangement by the Turkish Council of Higher Education in 2018 aimed to remove publications in predatory journals from the incentive allowance system. For this purpose, additional criteria have been introduced for international recognition of the journal. One of them is a minimum publication history of 5 years. However, these measures are not sufficient, and publications in these predatory journals and conferences should be removed completely from the scope of the incentive allowance system. To accomplish this will require diligent work because there are many low quality, non-predatory journals that are not included in major indices (Web of Science, Scopus, DOAJ etc.).

Universities in countries like Turkey must develop strategies to mitigate predatory publishing. One strategy adopted by several universities is that publications in those journals do not count toward academic evaluation or promotion. With this aim, in March 2019, the Turkish Council of Higher Education decided that scientific papers published in predatory journals would not be taken into account in academic promotion and assignment. Thus, Turkey has taken the step of becoming one of the first countries to implement this in the world. Since there is no agreed list of predatory journals and dubious conferences around the world, this process must be carefully implemented. Perhaps it is healthier to point out the good (white list: DOAJ, Science Citation Index, MEDLINE etc.) instead of the bad (blacklist).

Since the problem is international and complex, we need to know that it is not an easy solution. But a few months ago, there was a promising news from America. A publishing company was ordered by a federal court to pay $50.1 million to the Federal Trade Commission for deceptive and predatory publishing activities (12). This US federal court's decision against the questionable publishers was the first and encouraging in this regard. It should be noted, however, that the solution largely involves breaking the authors' demand for these questionable journals and conferences.

## Figures and Tables

**Table 1:**
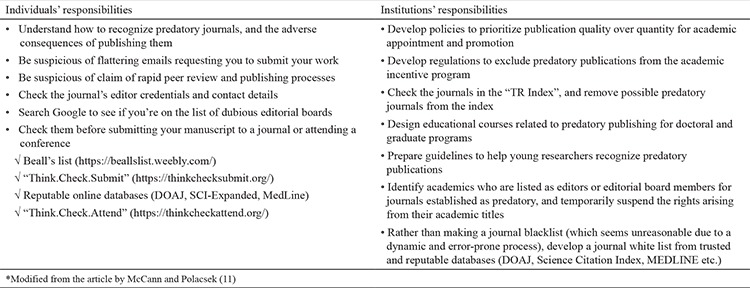
Recommendations for individuals and institutions to mitigate predatory publishing*
